# Planet Contamination with Chemical Compounds

**DOI:** 10.3390/molecules27051621

**Published:** 2022-03-01

**Authors:** Albert T. Lebedev, Susan D. Richardson

**Affiliations:** 1 Chemistry Department, M.V. Lomonosov Moscow State University, Leninskie Gory 1/3, 119991 Moscow, Russia; 2 Department of Chemistry & Biochemistry, University of South Carolina, Columbia, SC 29208, USA; richardson.susan@sc.edu

The number of known priority pollutants and emerging contaminants of environmental concern currently exceeds several thousand (US EPA Part 423, US EPA Part 401). It grows everyday as new pollutants enter the environment (NORMAN List of Emerging Substances). The number of chemical compounds registered in the Chemical Abstracts Service (CAS^®^) registry in 2020 reached 160 million [1]. Moreover, numerous reactions in the environment result in formation of many novel compounds, which do not yet have assigned CAS numbers. These products may possess higher toxicities than their parent compounds [2,3,4,5]. This expanding list can also include their metabolites and degradation products, as well as reaction products from numerous atmospheric and aquatic reactions. Therefore, it becomes more and more challenging to conduct comprehensive screening for known and emerging environmental contaminants. Today, various laboratories all over the world work on the identification and quantification of environmental pollutants and contaminants using more and more powerful approaches and analytical tools. In the majority of these studies, a targeted approach is used, when the investigators know, a priori, what they are trying to detect. Usually the hazardous compounds with confirmed toxicity (heavy metals, polycyclic aromatic hydrocarbons, polychlorinated biphenyls, etc.) are selected for monitoring, mapping, or estimation of their levels in response to some applied environmental strategies. An alternative, non-target approach involves attempts to identify all the chemical compounds in the samples [6,7,8,9,10,11,12]. The latter represents a challenging task, although it allows the establishment of novel contaminants and the discovery of pollution sources in different regions of the planet. On the basis of these studies, local lists of priority pollutants can be created, followed by the establishment of the sources of these pollutants and development of adequate policies to address those sources.

This special issue of Molecules, entitled “Planet contamination with chemical compounds” combines contributions from China, Colombia, Poland, Russia, Slovenia, Saudi Arabia, Egypt, and South Africa dealing with novel environmental approaches and specific case studies.

The comprehensive review of Kulikova and Perminova [13] deals with interactions of microorganisms and humic matter, representing the most complex mixture of natural organic compounds on our planet. The properties of the latter require detailed studies, as they may be beneficial for encapsulating heavy metals or can be undesirable by serving as the major source of disinfection by-products, formed during drinking water treatment. The properties of humic substances (HSs) are discussed in this review in terms of microbial utilization, degradation, and transformation. The data on biologically active individual compounds found in HSs, as well as the main bacteria and fungi species responsible for HS degradation and reduction, are summarized. Some promising aspects of interactions between microorganisms and HSs are discussed as a feasible basis for nature-like biotechnologies, including the production of enzymes capable of catalyzing the oxidative binding of organic pollutants to HSs, enhancement of organic pollutant biodegradation, and reduction of the formation of CH_4_ in temporarily anoxic systems. To facilitate the development of HS-based technologies, complex studies addressing these factors are in demand.

Non-target analysis with liquid chromatography (LC)-high resolution-mass spectrometry (MS) usually provides reliable information on the elemental composition of a contaminant. Nevertheless, elucidation of its structure presents a challenge. The idea of popularity/abundance of chemical compounds may be quite helpful for that purpose. To have a clear quantitative basis, obtained frequency distributions of chemical compounds over indicators of their popularity/abundance are discussed in the paper of Milman and Zhurkovich [14]. Popularity indicators are the number of information sources, the number of chemical vendors, counts of data records, and other variables assessed from two large databases, namely ChemSpider and PubChem, which are characteristic of the results of human/social activity. A relatively small group of the most popular compounds has been denoted, conventionally accounting for a few percent (several million) of compounds. These compounds are most often explored in scientific research and are practically used. Accordingly, popular compounds are typically considered as first analyte candidates for identification in non-target analysis.

The paper of Makhadi et al. [15] deals with the influence of municipal solid waste landfill sites to the groundwater quality in the city of Bloemfontein, South Africa. Samples from the two landfill sites located in two contrasting geological terrains were collected in the autumn and winter seasons to assess any possible seasonal variations. Targeted analyses involving physicochemical (pH, electrical conductivity, total dissolved solids, chemical oxygen demand, and total organic carbon) and microbiological parameters (*E. coli*, total coliform) were carried out. The results demonstrated the predominance of Ca, Mg, SO_4_, and HCO_3_ ions, and some of the parameters exceeded the South African National Standard (SANS241:2015) and World Health Organization (WHO) permissible limits for drinking water. Geological issues were found to play a notable role in the distribution of contaminants into the groundwater systems. The Northern landfill site demonstrated poorer water quality in comparison to the Southern landfill site on the basis of physicochemical parameters, while the latter showed higher microbial contamination due to elevated *E. coli* and total coliform concentrations.

The presence of 17 per- and polyfluoroalkyl substances (PFASs), as representatives of emerging contaminants in the Gulf of Gdansk, are discussed in the paper of Gałęzowska et al. [16]. Due to wide use of PFASs (e.g., in metal-plating, in fire-fighting foam, lubricants) and their resistance to degradation, they occur widely in the environment. Results of the chemical analysis, including the levels of the targeted compounds and sediment ecotoxicity, were used to assess environmental risk. Since for most sediments, toxic effects were not observed in the toxicity tests with *Heterocyprisinconguens* and *Aliivibrioficsheri*, the results of environmental risk assessment indicate that the target analytes did not generate high impact on the aquatic life so far.

Another paper from Poland [17] aimed to assess the levels of 98 multi-class target pharmaceuticals, including cardiovascular drugs, antidepressants, hypnotics, antibiotics, and sulfonamides, heavy metals and trace elements, as well as cholesterol and its 5 derivatives, occurring in the muscle tissues of fish caught in the Baltic Sea. The assessment of health hazards due to contamination is necessary to introduce national legislation and global standards aimed at reducing or even eliminating exposure to these contaminants. Based on the hazard factor for Hg, Pb, Cd, and Ni (target hazard quotient, THQ < 1), it was found that the consumption of the studied fish does not constitute a health risk. However, the THQ for As remained >1, indicating possible risk from that metal.

Slovenian research [18] investigated microplastics (MPs), their presence, routes of entry, and impacts on biota in the soil environment. Serious problems include the lack of standardized methods for their identification in environmental samples. The authors applied headspace-solid phase microextraction followed by gas chromatography (GC)–MS for the determination of polyethylene terephthalate, polystyrene, polyvinyl chloride, polyethylene, and polypropylene in environmental samples. The proposed method is based on the identification of compounds formed during the controlled melting process of specific coarse (1–5 mm) and fine fractions (1 mm–100 μm) of MPs. The method was upgraded for the identification of individual polymer types in plastic blends and in complex environmental matrices (soil and algae biomass).

The paper of Vozhdaeva et al. [19] throws light upon the problem of disinfection by-product (DBP) formation. It summarizes the results of almost 20-year monitoring of volatile and semi volatile DBPs in the drinking water of the city of Ufa, situated close to the border of Europe and Asia. The primary focus was placed on the brominated species, their sources, and mechanisms of formation. Although the prevalence of chlorine over bromine atoms in the structures of volatile DBPs is a well-known fact for most aqueous chlorination reactions, the situation with semi-volatile DBPs appeared to be reversed. Bromide in natural waters and hypobromite as an impurity in technical-grade sodium hypochlorite used for water disinfection are the main sources of bromine in DBPs. These results allowed the proposal of a new mechanism of formation for the organohalogenated species by fast penetration of bromine into the humic acid molecules and their oxidative destruction by active chlorine. A significant contribution of oxygen to the composition of semi-volatile compounds proves the decisive role of the dissolved organic matter oxidative destructive processes in the formation of organohalogenated species. Statistical analysis revealed notable linear correlations for trihalomethane and haloacetic acid formation vs. chlorine dose. On the contrary, halogenated semi-volatile products do not demonstrate any correlations with the water quality parameters or chlorine dose.

The toxicity of rocket fuel 1,1-dimethylhydrazine (UDMH) represents a serious problem, especially when it deals with the sensitive Arctic ecosystem. A group from Arkhangelsk University (Arkhangelsk, Russia) for several years has studied the fate of UDMH in the environment and identified numerous transformation products. Since the majority of these products are rather polar, classic liquid-liquid extraction followed by GC-MS often leads to rather poor results. Popov et al. [20] applied accelerated water sample preparation (AWASP) based on the complete removal of water with anhydrous sodium sulfate and transferring analytes into dichloromethane. This method ensured attaining near-quantitative extraction of 23 out of 29 transformation products in about 5 min. GC-MS with pyridine-d_5_ as an internal standard allowed for development of a rapid, simple, and low-cost method for simultaneous quantification of UDMH transformation products, with detection limits of 1–5 μg L^−1^ and linear range spanning 4 orders of magnitude. The method validation involved analysis of aqueous solutions of rocket fuel subjected to oxidation with atmospheric oxygen, as well as pyrolytic gasification in supercritical water, modeling wastewater from carrier rocket launch sites.

Joint research of investigators from Saudi Arabia and Egypt [21] investigates the leaching behavior of sulfamethoxazole (SMZ) through contaminated-manure-amended sandy loam soil at different pH levels. SMZ belongs to the sulfonamide antibiotic group and has been extensively consumed since the 1960’s for treatment of human and animal diseases. Vertical translocation/leaching of SMZ through manure-amended sandy loam soil, along with application of biochar for SMZ retention to prevent its penetration to the surface and ground water, were investigated in this study. The highest SMZ leaching was observed at pH 7.0 in the first 40 h, which lessened with lower pH, owing to the variation in SMZ speciation. Electrostatic interactions, H-bonding, and π-π electron donor acceptor interactions could rationalize the higher SMZ adsorption onto biochar. Jujube wood waste-derived biochar could serve as an efficient and cost-effective technology to reduce the mobility of SMZ in contaminated-manure amended soil.

Polish researchers [22] in their paper throw light upon the presence of toxic metals in eye shadow cosmetic products. Ninety-four products differing in numerous parameters were selected for this research. ICP-MS was used to determine the levels of Ag, Ba, Bi, Cd, Pb, Sr, and Tl. The spread of results was rather wide. For example, although some samples contained Cd levels below the LOQ, two of them contained levels exceeding its safe value. The safe level of Pb was also exceeded in one sample. Interestingly, concentrations of Cd and Pb were alarmingly high in some eye shadows intended for children. The highest concentration of Cd reached almost 4 mg/kg, and that of Pb-16 mg/kg. Big data analysis was carried out using PCA analysis. The presence of statistically significant differences was confirmed for all parameters, with an exception of the color of the eye shadow.

Plants may synthesize numerous compounds that reduce attacks of herbivores.Thus, rattlepods or genus *Crotalaria* (Fam. Fabaceae) plants produce pyrrolizidine alkaloids as a direct defense against generalist herbivores. In the paper of Colombian researchers [23], herbivore-induced plant volatiles emitted by *Crotalaria nitens* Kunth plants were studied by GC-MS. Compounds emitted by *C. nitens* were mainly green leaf volatile substances, terpenoids, aromatics, and aldoximes (isobutyraldoxime and 2-methylbutyraldoxime), with maximum emission six hours after the attack. It is worth mentioning that many of these compounds were not detected when the leaves suffered mechanical damage. Most compounds emitted by *C. nitens* leaves were reported to have ecological functions in other models of plant-insect interaction and even in plant–plant communication. Therefore, the *U. ornatrix* caterpillar induces a specific response in *C. nitens* plants, repelling conspecific moths from attacked plants and favoring oviposition in those without damage. The results definitely showed the importance of volatiles in plant–insect interactions.

This special issue, prepared by scientists from several countries throughout the world, illustrates the breadth of environmental research on chemical contaminants affecting our Planet.
